# The Validation of the Selective Mutism Questionnaire for Use in the Dutch Population

**DOI:** 10.1007/s10578-022-01387-8

**Published:** 2022-06-27

**Authors:** Chaya Rodrigues Pereira, Judith B. M. Ensink, Max G. Güldner, Kees Jan Kan, Maretha V. De Jonge, Ramón J. L. Lindauer, Elisabeth M. W. J. Utens

**Affiliations:** 1 Levvel, Academic Center for Child and Adolescent Psychiatry, Amsterdam, The Netherlands; 2 Amsterdam UMC, University of Amsterdam, Department of Child and Adolescent Psychiatry, Amsterdam Public Health, Amsterdam, The Netherlands; 3 Research Institute of Child Development and Education, University of Amsterdam, Amsterdam, The Netherlands; 4 Faculty of Social Sciences, Department of Education and Child Studies, Clinical Neuroscience and Developmental Disorders, University Leiden, Leiden, The Netherlands; 5 Department of Child and Adolescent Psychiatry/Psychology, Erasmus MC – Sophia Children’s Hospital, Rotterdam, The Netherlands

**Keywords:** Selective mutism, Selective mutism questionnaire, Psychometric, Parent report, Validation

## Abstract

Selective mutism (SM) is an anxiety disorder in children/adolescents, characterized by the absence of speaking in specific social situations, mostly at school. The selective mutism questionnaire (SMQ) is a parent report, internationally used to assess SM symptomatology and treatment outcomes. Since no assessment instrument for SM was available in the Netherlands, our aim was to investigate the psychometric properties of the Dutch translation of the SMQ, through reliability, confirmatory factor, and ROC analyses conducted on data obtained in 303 children (ages 3–17 years; clinical SM group n = 106, control group n = 197). The SMQ turned out to be highly reliable (α = 0.96 in the combined sample; 0.83 within the clinical group) and followed the expected factor structure. We conclude that the Dutch version of the SMQ is a reliable and valid tool both as a screening and clinical instrument to assess SM in Dutch speaking children.

## Introduction

Selective mutism (SM) is a relatively rare anxiety disorder (prevalence rates varying from 0.2% to 1.9% [1, 2]) that is characterized by consistent failure to speak in various specific social situations where speaking is expected (e.g., at school), whereas the child does speak in other situations (e.g., at home) [3]. SM typically manifests between the ages of 3 and 5, coinciding with the start of (pre)school and cannot be attributed to a language or speaking disorder [4, 5]. Parents may not always realize there is a problem, as the children do speak freely at home. In such cases, the consistent failure to speak needs to be noticed by professionals in the life of the child, for example at school [6–8]. A major problem in recognizing SM, is that there is a lack of validated instruments that assess the different responses associated with SM, and are able to distinguish children with SM from those with other anxiety disorders. Without adequate instruments, risk increases that SM is overlooked or not recognized as such [9]. If as a result no timely intervention is started, this can lead to chronic and complex anxiety and mood issues. Currently the selective mutism questionnaire (SMQ) is the most widely used screening and assessment tool with good psychometric properties, that is able to distinguish children with SM from other anxiety disorders [10]. Besides the SMQ, there are few other instruments investigating SM symptomatology; such as the Frankfurt Scale of Selective Mutism (FSSM [11]) or the Speech Situations Questionnaire (SpSQ [12]), however limited research has been conducted investigating their psychometric properties.

The SMQ assesses speaking behavior across different situations. It can be used by healthcare professionals in the first line of care (general practitioners, municipal child health clinics, school doctors and counselors). The psychometric studies into this instrument [10, 13–15] demonstrate a three factor structure consisting of school, home/family and public/social settings. Bergman et al. [10] first described the SMQ and initially studied the instrument in an internet sample of 589 participants (3–11 years) where parents identified their child as having difficulties in speaking in some settings. In their second study the psychometric properties of the SMQ were investigated in a group of 48 children (3–10 years) with SM and 18 anxious children without SM. Bergman et al. report internal consistency of Cronbach’s α = 0.84. In addition, other groups investigated the SMQ: The study of Letamendi et al. [13] included 102 parents of children with SM and 43 parents of children without SM (3 to 11 years), reporting Cronbach’s α = 0.783. In addition, the questionnaire was translated to Norwegian [14] and Spanish [15] showing good psychometric properties within these languages. Oerbeck et al. [14] investigated the psychometric properties of the SMQ in Norway, in 32 children with SM and 32 typically developing children (3–9 years), reporting Cronbach’s α = 0.96 in the total sample. Additionally, a study into a Spanish version of the SMQ by Olivares-Olivares et al. [15] included 110 children with SM (3–10 years), reporting Cronbach’s α = 0.90. Data from their Spanish sample fitted the factorial model of Bergman et al. [10], and their data on the reliability and validity of the Spanish SMQ were robust. Authors concluded that the Spanish SMQ is a good instrument for assessing SM in Spanish-speaking children.

The aim of this study is to facilitate early screening of SM in children by conducting a validation study of the translated version of the SMQ in the Netherlands. In contrast with previous studies investigating the psychometric studies of the SMQ, in our academic center for child and adolescent psychiatry we see a broad age range (3–18 years vs. 3–11 years in previous studies) of children and adolescents being referred for diagnostics and treatment due to presumable SM. This seems in line with a general increase of referral of older children to clinical practice, and growing attention for SM in older children. Therefore we decided to also include older participants as to provide a realistic representation of our population.

The validation of the Dutch SMQ has two purposes: firstly the ability of the instrument to screen for SM, secondly the ability to assess symptom level severity (which for example can be monitored following treatment).

## Methods

### Participants

Among the children who participated in this study (n = 303), 54% were female and 46% were male. The age of the children ranged from 3 to 17 years with a mean age of 7.94 (SD = 3.84). Of the total group, 27.3% was bilingual. The clinical group consisted of 106 children (age range 3–16 years, mean = 6.33, SD = 2.98). 42.5% of the children in this group were boys, 57.5% were girls. In addition, 45.2% of the clinical group was bilingual or multilingual. The control group consisted of 197 children with no SM classification or SM related problems (age range 3–17 years, mean = 8.8, SD = 3.98). 48% of the children in this group were boys and 52% girls. Furthermore, 17.8% of the control group was bilingual or multilingual. The education level of the families in the clinical group and the control group is shown in Table 1.

Despite our efforts to include a representative control group in terms of gender, age and bilingualism, especially parents of younger children (< 4 years) and bilingual/multilingual families were less likely to participate, therefore our clinical group was younger, and conducted more children with bilingual/multilingual background in comparison with our control group. Furthermore, the education level of parents in the control group was higher than in the clinical group (See Table 1).

### Procedure

Within the clinical group, all children were referred to our academic center for child and adolescent psychiatry due to suspected SM and were referred for treatment and/or diagnostics. Some of them (N = 83), participated in an ongoing randomized controlled trial (RCT) study [16]. The data collection of the clinical group was part of care as usual and was also performed in the context of the baseline assessment in the RCT [16]. All assessments were performed by psychologists of the SM expertise team of our institution. To recruit a control group with similar age and gender distribution, (pre)elementary schools, secondary schools, sports and recreation clubs in Amsterdam and surrounding areas were contacted to distribute information about the research project. The participants in the control group responded to posters, over 6500 folders and information that was distributed in (newsletters of) over 100 (pre)schools, around 70 sports clubs and other recreation associations such as music schools in the same regional areas as the clinical group. Parents of 270 children were interested in participating and filled out a contact form and received further information and informed consent forms. After receiving information, N = 197 families decided to participate in the control group. Parents and children from age 12 and older filled out a written informed consent, questionnaires were sent out through a secured online program and a telephone interview for additional measures similar to our clinical group was performed by research psychologists and master students under supervision of a psychologist of the SM expertise team. In both groups, demographic data were collected with use of a semi structured interview (e.g., gender, age, bilingualism of the child, parental educational and occupational status, nationality of child and parents and languages being spoken at home).

The study was approved by the Medical Ethical Committee of the Amsterdam University Medical Center.

### SMQ

#### SMQ

The SMQ [10] is a parent reported questionnaire, assessing the child’s speaking behavior and SM symptoms in various situations. The SMQ consists of two scales: the symptom scale (17 items) stating different situations in which a child is expected to speak, covering three domains: school (6 items, e.g., “When appropriate, my child asks his or her teacher questions.”), family (6 items, e.g., “When appropriate, my child talks to family members while in unfamiliar places.”) and social/public situations (5 items, e.g., “When appropriate, my child speaks with his or her doctor and/or dentist.”). Parents rate the frequency of speaking behavior on each item using a 4-point scale (3 = always, 2 = often, 1 = seldom and 0 = never). An SMQ symptom scale score was calculated as an individual’s average item score multiplied by the number of items (17), equaling the sum score in case no data are missing. SMQ symptom scale scores thus ranged from a minimum of 0 to a maximum of 51; the lower the score on the SMQ symptom scale the more problems with daring to speak and the less speaking behavior. In order to realize a cutoff score for the screening of SM, the SMQ scoring in this study was converted so that a higher score indicates more problems.

The interference scale includes 6 items (e.g., “How much does not talking interfere with school for your child?”, response categories: “no”, “slightly”, “moderately” and “extremely”). An SMQ interference scale score was again calculated as an individual’s average item score multiplied by the number of items (6). A higher score on the SMQ interference scale indicates higher impact on child and family functioning.

### Additional measures

#### ADIS-C

The Anxiety Disorders Interview Schedule for Children for DSM-IV [17] is a semi structured diagnostic interview to assess anxiety and mood disorders according to DSM-IV criteria in children and adolescents. The SM segment of the ADIS-C was conducted in parents, and the child version was conducted in children from ages 8 and up, if they agreed to answer the questions. The SM segment consists of 8 items, covering the speaking behavior and school functioning of the child. Parents rate the interference of the symptoms with the child’s daily life on a 9 point scale (0–8). The interviewer rates the interference on the same 9 point scale for the Clinician Severity Rating (CSR). A cutoff of 4 on the CSR indicates a classification. The ADIS-C was part of the care as usual in the clinical sample, and the SM segment was administered through a phone interview in the control group. If the parent interference rating and the CSR differed, the CSR was decisive.

#### Children’s Internalizing and Externalizing Problems

The Child Behavior Checklist (CBCL) [18, 19], preschool and school-age versions, is a parent report questionnaire assessing behavioral and emotional problems in children. The Youth Self Report (YSR) [18] is the parallel questionnaire of the CBCL, formulated for the child from ages 11 and older. The CBCL and the YSR have different subscales, combining in an internalizing scale (includes Withdrawn, Somatic Complaints, and Anxiety/Depressed Problems) and externalizing scale (includes Delinquent and Aggressive Behaviors). T-scores of 65 and higher are in the clinical range.

### Statistical Analysis for Validation

As mentioned in the introduction, the validation of the SMQ consisted of two parts. The first part concerned an investigation of the descriptive statistics, followed by the psychometric properties of the SMQ when using the instrument as a screening device. To this end, we carried out a reliability analysis (obtaining Cronbach’s α), a receiver operating characteristic (ROC) curve analysis, and a (confirmatory) factor analysis on the correlational structure among the 17 items of the SMQ in the subjects sample as whole, i.e., the combined clinical and control group. Following the factor analysis, the discriminant validity between the SMQ and CBCL was established. The second part concerned an investigation of the SMQ as a clinical measuring instrument. To this end, we repeated the reliability and factor analysis within the clinical subsample only, while also considering the reliability and validity of the interference scale.

## Results

### SMQ

The overall sample mean on the SMQ total score for the symptom scale was 32.88 (SD = 14.52) As shown in Table 1, there was a significant difference in the SMQ scores on the symptom scale of the clinical group (mean = 15.06, SD = 6.88) and the control group (mean = 41.24, SD = 8.27).

**Table 1:** Descriptives

	SM group (N = 106)	Control group (N = 197)
Age	Mean = 6.33, SD = 2.98	Mean = 8.80, SD = 3.98**
Gender	42.5% boys, 57.5% girls	48% boys, 52% girls
Languages	45.2% bilingual or multilingual	17.9% bilingual or multilingual**
Parents’ education level^a^	Mothers: 41.5% no higher education, 58.5% higher education Fathers: 51.9% no higher education, 41.5% higher education, 6.6% absent	Mothers**: 5.1% no higher education, 94.9% higher education Fathers**: 24.5% no higher education, 73.0% higher education, 2.5% absent
*SMQ*		
Symptom scale	Mean = 15.06, SD = 6.88	Mean = 41.24, SD = 8.27**
Interference scale	Mean = 10.18, SD = 3.63	Mean = 0.56, SD = 1.88**
*CBCL (t-scores)*		
Internalizing scale	Mean = 61.06, SD = 9.63	Mean = 45.94, SD = 9.64**
Externalizing scale	Mean = 48.34, SD = 10.49	Mean = 42.46, SD = 7.40**
Total scale	Mean = 54.63, SD = 10.24	Mean = 43.04, SD = 8.63**
*YSR (t-scores)*		
Internalizing scale	Mean = 52.46, SD = 11.26	Mean = 47.83, SD = 9.72
Externalizing scale	Mean = 40.54, SD = 8.60	Mean = 44.83, SD = 8.35
Total scale	Mean = 46.46, SD = 10.33	Mean = 46.78, SD = 9.08

SMQ = Selective Mutism Questionnaire, CBCL = Child Behavior Checklist, YSR = Youth Self Report

**Significant group differences (p < 0.05)

^a^No higher education: no education, primary education, general secondary education, lower or middle vocational education, pre-university education; higher education: higher vocational education, university

### CBCL and YSR Scores

There was a significant difference in the CBCL scores for the total internalizing scale (clinical group: mean = 61.06, SD = 9.63, control group: mean = 45.94, SD = 9.64), the total externalizing scale (clinical group: mean = 48.34, SD = 10.49, control group: mean = 42.46, SD = 7.40) and the total scale (clinical group: mean = 54.63, SD = 10.24, control group: mean = 43.04, SD = 8.63) between the clinical group and the control group (see Table 1).

In the clinical group, 13 children completed the YSR, and in the control group 60 children did. Due to small samples no conclusions can be drawn. For descriptive purposes, we report means and standard deviations in Table 1 for the total internalizing scale (clinical group: mean = 52.46, SD = 11.26, control group: mean = 47.83, SD = 9.72), the total externalizing scale (clinical group: mean = 40.54, SD = 8.60, control group: mean = 44.83, SD = 8.35) and the total scale (clinical group: mean = 46.46, SD = 10.33, control group: mean = 46.78, SD = 9.08).

## Validation of the SMQ as a Screening Instrument

### Reliability Analysis

The analyses of the SMQ as a screening instrument were conducted on the data of the total combined sample. As part of that analysis we first conducted an item analysis in which we obtained the Cronbach’s alpha coefficient of the 17 item scale. This coefficient was 0.96 (95% confidence interval = 0.95, 0.97), indicating high internal consistency reliability. Tables 2 and 3 provide more detailed results of this analysis, from which it can be obtained that with the exception of item 2, all items contributed to the internal consistency reliability.

**Table 2:** Item means, standard deviations and inter correlations of the SMQ symptom scale

Item	Item 1	Item 2	Item 3	Item 4	Item 5	Item 6	Item 7	Item 8	Item 9	Item 10	Item 11	Item 12	Item 13	Item 14	Item 15	Item 16	Item 17
Item 1	1.00																
Item 2	0.13	1.00															
Item 3	0.79	0.23	1.00														
Item 4	0.83	0.17	0.75	1.00													
Item 5	0.86	0.15	0.82	0.87	1.00												
Item 6	0.80	0.16	0.76	0.83	0.87	1.00											
Item 7	0.64	0.16	0.66	0.60	0.61	0.57	1.00										
Item 8	0.63	0.14	0.62	0.57	0.62	0.58	0.72	1.00									
Item 9	0.68	0.04	0.68	0.60	0.67	0.62	0.62	0.63	1.00								
Item 10	0.39	− 0.04	0.36	0.37	0.34	0.38	0.42	0.51	0.48	1.00							
Item 11	0.66	0.05	0.63	0.62	0.65	0.60	0.65	0.64	0.76	0.67	1.00						
Item 12	0.62	0.06	0.64	0.60	0.63	0.58	0.68	0.54	0.65	0.50	0.79	1.00					
Item 13	0.65	0.04	0.65	0.67	0.74	0.68	0.57	0.58	0.64	0.45	0.69	0.66	1.00				
Item 14	0.72	0.08	0.69	0.69	0.78	0.70	0.59	0.59	0.66	0.45	0.70	0.66	0.83	1.00			
Item 15	0.74	0.13	0.75	0.73	0.80	0.76	0.59	0.60	0.69	0.55	0.73	0.66	0.74	0.80	1.00		
Item 16	0.72	0.08	0.73	0.74	0.81	0.77	0.59	0.60	0.70	0.52	0.71	0.63	0.75	0.81	0.87	1.00	
Item 17	0.74	0.14	0.85	0.75	0.82	0.77	0.68	0.63	0.73	0.45	0.69	0.71	0.78	0.76	0.81	0.82	1.00
Mean	1.90	1.69	2.11	1.62	1.62	1.62	2.54	2.46	2.36	2.36	2.19	2.61	1.49	1.53	1.77	1.46	1.84
SD	1.14	1.15	1.15	1.16	1.22	1.17	0.77	0.82	0.86	0.93	1.02	0.81	1.12	1.14	1.21	1.17	1.17

Analysis of the SMQ symptom scale in the total sample (clinical group + control group): item means, standard deviations and inter correlations of each item

**Table 3:** Item characteristics of the SMQ; number of valid responses (n), item correlations with sum score (r) and Cronbach’s alpha when the item is dropped (α)

Item	n	r	α
Item 1	283	0.84	0.96
Item 2	285	0.27	0.97
Item 3	285	0.83	0.96
Item 4	284	0.88	0.96
Item 5	283	0.92	0.96
Item 6	279	0.88	0.96
Item 7	284	0.72	0.96
Item 8	285	0.72	0.96
Item 9	276	0.78	0.96
Item 10	281	0.56	0.96
Item 11	284	0.81	0.96
Item 12	152	0.78	0.96
Item 13	284	0.84	0.96
Item 14	283	0.87	0.96
Item 15	283	0.89	0.96
Item 16	282	0.89	0.96
Item 17	278	0.92	0.96

Item characteristics of the SMQ symptom scale in the total sample (clinical group + control group)

n = number of valid responses

r = item correlation with sum score on the SMQ symptom scale

α = Cronbach’s alpha when the item is dropped

### ROC Curve Analysis

**Fig. 1:**
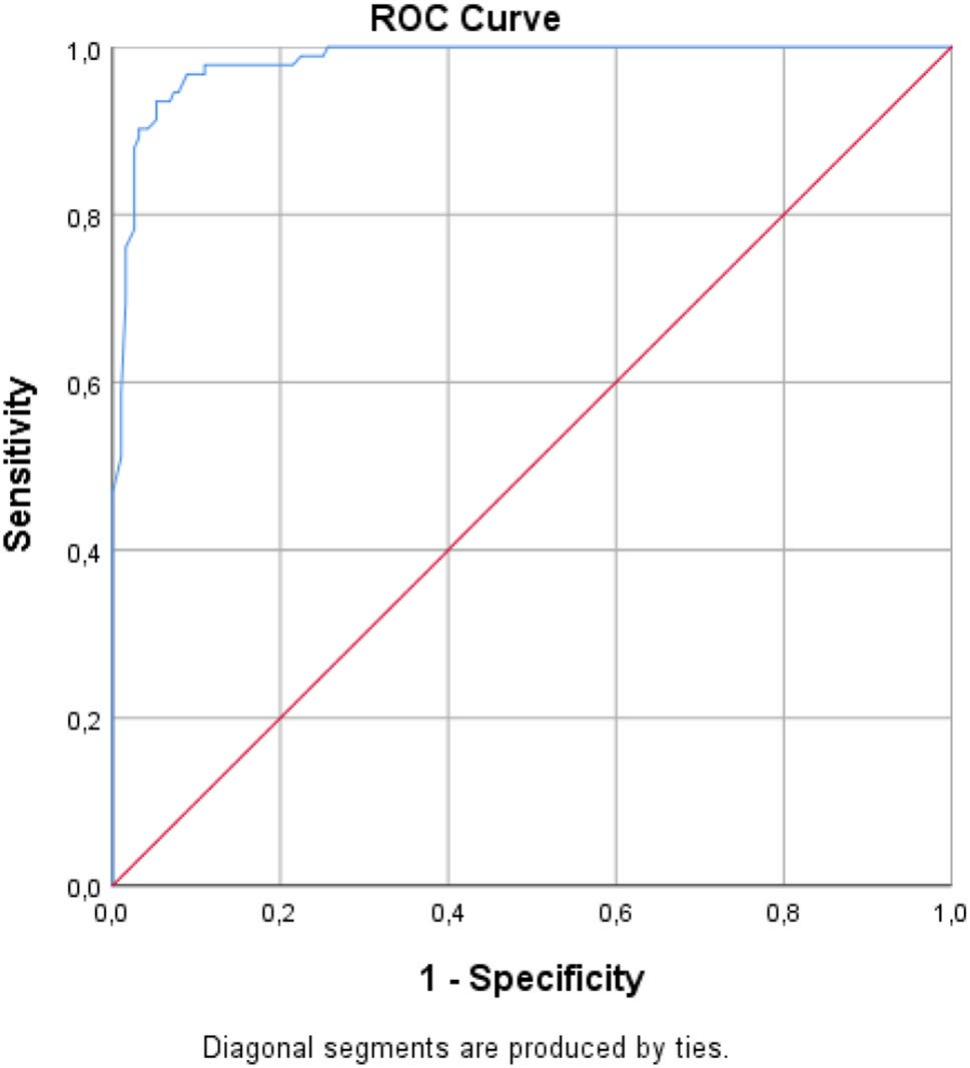
ROC analysis of SMQ with optimal cutoff values

In general, an ROC analysis refers to the analysis of the diagnostic capacity of a (binary) classifier system. The sensitivity (true positive rate) is plotted against the false positive while varying a threshold or cutoff score. In order to realize a cutoff score for the screening of SM, the SMQ scoring is converted so that a higher score indicates more problems. Figure 1 provides the ROC curve of the SMQ. Full results are shown in Table 4. Optimal cutoff values were evaluated based on ROC curve analysis. The symptom scale of the SMQ showed satisfactory discriminating properties in differentiating participants with and without SM classification. The SM classification was determined with the use of the ADIS-C SM segment. The ROC analysis showed a significant result with area under the curve (AUC) = 0.982 (95% confidence interval = 0.97–0.99). With a cutoff score of 13, the sensitivity would be 100%, and the specificity would be 74.3%, as shown in Table 5.

**Table 4:** Coordinates of the ROC curve

Coordinates of the curve		
Test Result Variable(s): Mean SMQ Score × 17		
Positive if Greater Than or Equal To^a^	Sensitivity	1 − Specificity
− 1.0000	1.000	1.000
0.5000	1.000	0.901
1.0313	1.000	0.880
1.5313	1.000	0.874
2.0625	1.000	0.843
2.5625	1.000	0.827
3.0938	1.000	0.785
3.2938	1.000	0.749
3.7000	1.000	0.738
4.1250	1.000	0.681
4.6250	1.000	0.660
5.1563	1.000	0.634
5.6563	1.000	0.597
6.1875	1.000	0.555
6.5875	1.000	0.539
6.9000	1.000	0.529
7.2188	1.000	0.492
7.7188	1.000	0.487
8.2500	1.000	0.461
8.7500	1.000	0.450
9.0333	1.000	0.435
9.3146	1.000	0.429
9.7813	1.000	0.419
10.1000	1.000	0.387
10.4125	1.000	0.382
10.8125	1.000	0.377
11.1667	1.000	0.361
11.5104	1.000	0.356
11.8438	1.000	0.319
12.3750	1.000	0.298
12.8750	1.000	0.277
13.3000	1.000	0.257
13.8000	0.989	0.251
14.3667	0.989	0.241
14.8042	0.989	0.236
14.9375	0.989	0.225
15.4688	0.978	0.215
15.9688	0.978	0.194
16.5000	0.978	0.173
17.5000	0.978	0.136
18.0313	0.978	0.120
18.5938	0.978	0.115
19.1958	0.978	0.110
19.6333	0.967	0.110
20.0938	0.967	0.105
20.5938	0.967	0.089
21.6563	0.957	0.084
22.4896	0.946	0.079
22.8333	0.946	0.073
23.1875	0.935	0.068
23.5875	0.935	0.052
24.4000	0.924	0.052
25.2500	0.913	0.052
25.7500	0.902	0.042
26.2813	0.902	0.037
27.0938	0.902	0.031
28.1563	0.891	0.031
29.2188	0.880	0.026
29.8750	0.848	0.026
30.3000	0.826	0.026
30.7063	0.815	0.026
30.9063	0.783	0.026
31.4375	0.772	0.021
31.9375	0.761	0.016
32.4333	0.739	0.016
32.9021	0.728	0.016
32.9688	0.707	0.016
33.5000	0.696	0.016
34.5000	0.587	0.010
35.0313	0.543	0.010
35.5313	0.522	0.010
36.0625	0.511	0.010
36.6563	0.467	0.000
37.2938	0.424	0.000
37.7000	0.402	0.000
38.1250	0.391	0.000
38.6250	0.337	0.000
39.1563	0.326	0.000
39.4896	0.304	0.000
40.0208	0.283	0.000
40.6875	0.250	0.000
41.2188	0.228	0.000
41.7188	0.185	0.000
42.2500	0.174	0.000
42.7500	0.152	0.000
43.8125	0.141	0.000
44.8125	0.087	0.000
45.3438	0.054	0.000
46.7500	0.033	0.000
47.9063	0.022	0.000
49.0000	0.000	0.000

ROC curve analysis of the SMQ symptom scale sum score in the total group (clinical group + control group)

**Table 5:** ROC analysis of SMQ with optimal cutoff values

Group comparison	Optimal cutoff	Cutoff SE_100_	Cutoff SP_100_	ROC-AUC [95% CI]	Sensitivity^^^	Specificity^^^
Clinical vs. control	13	13.3	36.7	0.982** [0.970–0.994]	1.00	0.74

*SE*_*100*_ sensitivity of 100%, *SP*_*100*_ specificity of 100%, *AUC* area under the curve

^At optimal cutoff; **significant at p < 0.001

### Confirmatory Factor Analysis

Following previous validation studies into the SMQ [10, 13–15] we fitted an oblique (confirmatory) three factor model on the data of the total combined sample: scores on items 1 to 6 were regressed on a latent variable interpreted as mutism in the school context, scores on items 7 to 12 on a latent variable interpreted as mutism in the home context, and scores on items 13 to 17 on a latent variable interpreted as mutism in the social or public context. Age was included as a covariate i.e., as a predictor of all 17 items. Gender was also considered to be a possible covariate, but since correlations with gender were generally insignificant gender was eventually not included.

To judge the fit we used the often used criteria provided by Schermelleh-Engel, Moosbrugger and Müller [20] and reported the χ^2^ statistics on which these are based. We considered a model as acceptable when the value of the Comparative Fit Index (CFI) and Tucker-Lewis Index (TLI) were 0.95 or higher or Root Mean Square Error of Approximation (RMSEA) values lower than 0.08. Good fit was defined as CFI and TLI values greater than 0.97 and RMSEA values lower than 0.05.

According to most of these criteria, the theoretical model did not fit well (χ^2^ (101) = 323.162, p < 0.001; CFI = 0.955; TLI = 0.939; RMSEA = 0.085). However, adding a single residual correlation between items 13 and 14 improved the fit substantially and resulted in an acceptable fit (χ^2^ (100) = 278.945, p < 0.001; CFI = 0.963; TLI = 0.950; RMSEA = 0.077). We therefore concluded that the three factor structure was tenable. The factor loadings within the adapted model were generally high (median standardized loading: 0.839, see Table 6). This also held for the correlations between the three factors. We excluded item 2: “When appropriate, my child talks to selected peers (his/her friends) at school”. This item was dropped due to low inter-item correlation (see Table 2).

**Table 6:** Item loadings on the three SMQ factors and age effects in the total group

Item	School	Home	Social	Age
Item 1	0.843			0.158
Item 3	0.774			0.350
Item 4	0.899			0.195
Item 5	0.925			0.257
Item 6	0.882			0.277
Item 7		0.793		0.066
Item 8		0.745		0.156
Item 9		0.817		0.177
Item 10		0.586		0.154
Item 11		0.837		0.181
Item 12		0.858		0.145
Item 13			0.792	0.261
Item 14			0.836	0.264
Item 15			0.841	0.338
Item 16			0.868	0.307
Item 17			0.871	0.314

Factor intercorrelations			
	School	Home	Social
School	1		
Home	0.794	1	
Social	0.921	0.885	1

The confirmative factor analysis of the SMQ symptom scale yielded a 16-item solution consisting of three factors: school, home and social situations. The table shows the item loadings and factor intercorrelations on the three factors, and the age effects per item in the total group (clinical group + control group)

As a next step, the adapted three factor model (including covariate age) was rewritten as a higher order factor model, in which the three latent variables School, Home and Social context loaded on a general SM factor. This allowed for an investigation of the discriminant validity by regressing criterion variables—the normed CBCL internalizing, externalizing and total score—on the general SM factor.

Table 7 provides the results. From this table one can obtain that SM correlates moderately with those variables, but cannot be considered identical to any of these, corroborating previous empirical evidence in support of the interpretation of SM as a distinct disorder.

**Table 7:** Correlation of CBCL scales with SMQ in total group

CBCL scale	Correlation with SMQ
Internalizing	− 0.697
Externalizing	− 0.309
Total	− 0.576

Correlation of the CBCL scales with the SMQ symptom scale sum score in the total group (clinical group + control group)

## Validation of the SMQ as a Clinical Instrument

### Reliability Analysis

Within the clinical sample, the SMQ also showed good reliability; Cronbach’s alpha of the SMQ was 0.83 (95% confidence interval = 0.78–0.87). The additional 6 items that assessed interference displayed good reliability as well; Cronbach’s alpha was 0.81 (95% confidence interval = 0.76–0.87) and all items contributed.

### Confirmatory Factor Analysis

We repeated fitting the oblique (confirmatory) three factor model—including the two residual correlations—on the data of the clinical group only. As can be expected, the restriction of range lowered the factor loadings, the inter correlations between the factors (see Table 8), and the correlations with the CBCL scores (see Table 9). Nevertheless, the loadings were still substantial. The total SMQ score still correlated significantly and moderately with internalizing, but not with externalizing or total CBCL score.

**Table 8:** Item loadings on the three SMQ factors and age effects in the clinical group

Item	School	Home	Social	Age
Item 1	0.720			0.025
Item 3	0.441			0.224
Item 4	0.612			0.048
Item 5	0.740			− 0.060
Item 6	0.578			0.275
Item 7		0.695		− 0.144
Item 8		0.605		0.053
Item 9		0.689		− 0.018
Item 10		0.540		0.017
Item 11		0.714		− 0.154
Item 12		0.630		− 0.191
Item 13			0.778	0.152
Item 14			0.686	0.013
Item 15			0.578	0.191
Item 16			0.694	0.158
Item 17			0.590	

Factor intercorrelations			
	School	Home	Social
School	1		
Home	0.183	1	
Social	0.469	0.714	1

The confirmative factor analysis of the SMQ symptom scale yielded a 16-item solution consisting of three factors: school, home and social situations. The table shows the item loadings and factor intercorrelations on the three factors, and the age effects per item in the clinical group

**Table 9:** Correlation of CBCL scales with SMQ in clinical group

CBCL scale	Correlation with SMQ
Internalizing	− 0.450*
Externalizing	0.040
Total	− 0.173

Correlation of the CBCL scales with the SMQ symptom scale sum score in the clinical group

## Discussion

The results of the current study show the validity of the Dutch SMQ both as a screening tool and as a clinical instrument. The psychometric properties of the SMQ can be considered as good. First of all its consistency and reliability is high. Secondly, the instrument is able to distinguish well between individuals that fulfill the diagnostic criteria of SM and those that do not. Thirdly, the factorial structure of the SMQ was confirmed providing additional evidence for the validity of three subscales (Home/Family, School and Social situations), as already found by Bergman et al. [10]. Lastly, in line with findings from previous research, as mentioned in Bergman et al. [10], the SMQ shows that SM is a construct that differs from other variables, including externalizing and internalizing behavior as assessed by the CBCL.

Our results support earlier findings by Letamendi et al. [13] showing correlations between the internalizing scale of the CBCL and the SMQ. The correlations are not so high that both instruments seem to measure the same construct, thus both instruments can be regarded as measuring distinct constructs [21]. In addition, since the CBCL does not measure SM specifically, there is still a need for an instrument aimed to the speaking behavior of the child and the interference of SM in the daily life. This result corroborates again previous empirical evidence in support of the interpretation of SM as a distinct anxiety disorder [22].

In previous psychometric studies on the SMQ, no cutoff score was established. To enable low threshold access to care, we decided to investigate the possibility of using a cutoff score in our sample. To be able to calculate the cutoff score, we reversed the scoring of Bergman et al. [10] so that a higher score indicates more SM symptomatology to facilitate the use and interpretability. If a cutoff score of 13 would be used for the SMQ in the current sample, all children with SM would be screened as positive. Using such a sensitive instrument would ensure early detection. We recommend that for screening sensitivity ought to be prioritized over specificity, since the risk of missing a false negative and thus not referring for adequate treatment should be minimized in order to promote early detection and intervention. Since we know that early intervention improves prognosis, this would be preferable [7, 8]. In future research, this cutoff, based on reversed scoring, can be investigated in other samples to improve the generalizability of this finding and its use in clinical practice. If the original scoring of Bergman et al. [10] (higher score indicates less SM symptomatology and more speaking behavior) is maintained, we expect a cutoff score of 38 (51; maximum total score—13; cutoff with reversed scoring) where it is to be interpreted that a score of 38 or less is an indication for SM.

### Strengths and Limitations

A strength of this study was that we made an effort to recruit participants for the control group widely, spreading over 6500 folders, and involving over 100 (pre)schools and 70 sports and leisure clubs in different neighborhoods and zip codes of Amsterdam and its suburbs. By reaching out through social media, a radio interview and school newsletters, we intended to reach a diverse group. Furthermore a strength was that all children in our clinical group were referred to our specialized care center, thereby reflecting our regular clinical population. At our institution, we see children and adolescents from ages up to 18 and in some cases 23. To ensure that the instrument was applicable to our broad clinical population, another strength was that we were able to include also adolescents in our study. As previous psychometric studies included younger children [10, 13–15], the current broad age range in the sample is rather unique. Since SM is often underrecognized, which can result in misdiagnoses and delay in start of treatment [23], it was of surplus value that this study encompassed a broader age range both for our clinical population and the control group. This decreased the possibility that there were children with ‘latent’ SM in the control group, where the problem might not have been recognized at a younger age. An important innovative strength was that this study established cut-offs for screening purposes.

Among the limitations of this study is that the control group consisted of families that wanted to participate in the study intrinsically, and thereby possibly differed (selection bias) from the complete general population and the group that is referred to our institution for SM. The need to actively reach out to participate can lead to inevitable selection bias. Despite our efforts to include a representative typically developing control group, both groups differed significantly in terms of age and bilingualism. Even though we did actively distribute information in preschools and early childhood health centers, the participants in the control group were on average significantly older than the clinical group, which can have influenced the results. Noteworthy, however, both the clinical and the control group covered children in the preschool, elementary school and secondary school age groups. For future research, we recommend that the validity of the SMQ is further studied in older children and adolescents with SM.

In the current study we only included parent report measures (SMQ, ADIS and CBCL). Due to the small numbers of patients completing self-reports (YSR), due to the age-range of this instrument, no warranted statistical analyses on YSR data could be performed. For future research we recommend to study SMQ and YSR data for a larger group of clinical patients. In addition, we recommend also including data on the SMQ and also the TRF from teachers and/or schools as to include the perspective of the school where the not speaking is most present [24].

Now that the SMQ is validated for use in the Dutch population, it can be easily accessed by health care professionals. We conclude that the SMQ can be used as a reliable and valid screening tool to assess the need for further diagnostics. With high sensitivity and specificity it is a suited tool for clinical practice and research purposes. In the future the SMQ may be added to the student tracking system as an optional questionnaire when the school suspects the student might not talk as much in the school setting. The SMQ can be embedded in the Dutch system of first line care, through early childhood health centers and/or school and youth doctors. This would enhance the early detection of SM, thus improving the timely diagnosis and early start of treatment, leading to a better prognosis.

## Summary

Selective mutism (SM) is an anxiety disorder in children/adolescents, characterized by the absence of speaking in specific social situations, mostly at school. The Selective Mutism Questionnaire (SMQ) is a parent report, internationally used to assess SM symptomatology and treatment outcomes [10]. The present study investigated the psychometric properties of the Dutch translation of the SMQ, through reliability, confirmatory factor, and ROC analyses, using data of 106 clinical children with SM and 197 control children without SM. Results showed that the Dutch SMQ is highly reliable (α = 0.96 in the combined sample; 0.81 within the clinical group) and followed the expected factor structure. We conclude that the Dutch SMQ is a reliable and valid tool both as a screening and clinical instrument. Now that the SMQ is validated for use in the Dutch population, it can be easily accessed by health care professionals. The SMQ can be embedded in the Dutch system of first line care, through early childhood health centers and/or school and youth doctors. This would enhance the early detection of SM, thus improving the timely diagnosis and early start of treatment, leading to a better prognosis.
